# RDA-YOLO: A robust dynamic adaptive network for tiny insulator defect detection

**DOI:** 10.1371/journal.pone.0348869

**Published:** 2026-05-15

**Authors:** Xiaoxiong Zhou, Junchi He, Cheng Cheng, Guangming Zhang

**Affiliations:** 1 College of Electrical Engineering and Control Science, Nanjing Tech University, Nanjing, China; 2 Distribution Network Operation and Maintenance Center, State Grid Jiangsu Electric Power Co., Ltd., Guanyun County Electric Power Supply Company, Lianyungang, China; Swiss Federal Technology Institute of Lausanne, SWITZERLAND

## Abstract

Insulator defect detection is a critical component in ensuring the safe operation of smart grids. To achieve more effective detection, image-based inspection utilising drone aerial photography offers advantages such as low cost, high efficiency, and superior accuracy. Compared to other approaches, the You Only Look Once (YOLO) method demonstrates outstanding performance in insulator defect detection. However, it struggles to achieve satisfactory results when detecting small defects against complex backgrounds. To address this issue, this paper proposes a high-precision insulator defect detection algorithm named RDA-YOLO, which builds upon the YOLOv8 algorithm as its baseline model. Firstly, a reverse large-selection kernel module is designed to effectively adjust the receptive field size, enhancing feature extraction capabilities for long insulator strings and minute features. Secondly, a Dynamic Head replaces the original detection head, utilising its unified attention mechanism to obtain more consistent classification and localisation features. Finally, a distribution-aware Wise-IoU metric is proposed, modelling bounding boxes as two-dimensional Gaussian distributions. By employing normalised Wasserstein distance, this enhances the network’s detection capability for small targets. Experiments on a proprietary dataset demonstrate that, with only a modest increase in computational overhead, this network achieves 91.6% precision and 91.4% mAP0.5, outperforming other state-of-the-art algorithms. Moreover, we conducted extensive robustness experiments, which demonstrated that our approach achieves significantly enhanced robustness compared to baseline models, rendering it more suitable for detecting extreme weather conditions.

## Introduction

Insulators constitute one of the most numerous yet most failure-prone electrical components in transmission lines, their condition directly determining the stability of grid operation [1,2]. However, the majority of insulators, being perpetually exposed to the elements and subjected to various adverse weather conditions, are highly susceptible to developing defects. This frequently results in widespread grid incidents causing substantial economic losses [3]. Thus, routine and accurate checking of insulators is a necessary precaution towards the safe operation of power systems.

Insulator failure in high-voltage transmission systems is typically caused by a variety of physical mechanisms, including flashover, partial discharge and dielectric breakdown, amongst others. Surface flashover usually occurs on the surface of the insulator, triggered by the combined presence of contaminants and moisture, which leads to arc discharge. Partial discharge, on the other hand, refers to discharge that does not fully penetrate the insulator; over time, its prolonged effect can impair the performance of the insulator material. Dielectric breakdown refers to the complete loss of an insulator’s performance under the influence of a high electric field generated by high voltage. Although these mechanisms differ in physical or chemical terms, they often exhibit common characteristics in imaging, such as small scale, low contrast and irregular morphology. These failure mechanisms manifest as observable surface defects in images, and these defects can explain the origin of insulator defects at a physical level.

**Fig 1:**
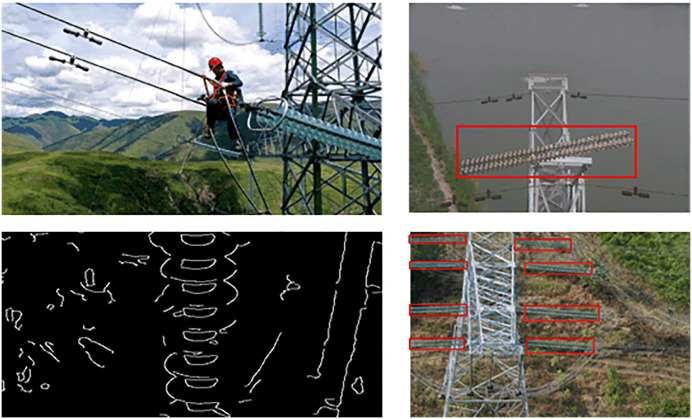
Comparison of advantages and disadvantages between traditional detection and deep learning detection. (This figure is generated based on the publicly available UPID dataset.).

As shown in Fig 1, early insulator defect detection primarily relied on manual inspection. However, such approaches are constrained by complex terrain conditions and suffer from low efficiency and high operational risk, making them unsuitable for large-scale and long-term deployment. In recent years, with the rapid development of unmanned aerial vehicle (UAV) technology, aerial inspection based on drone imagery has gradually replaced manual inspection due to its advantages in cost-effectiveness, safety, and operational flexibility.

In the early stage of automated inspection, traditional image processing methods, such as edge detection and segmentation techniques, were widely used for insulator and defect detection. Although these methods can achieve satisfactory performance under controlled conditions, they exhibit limited robustness when dealing with complex backgrounds and varying environmental conditions.

With the advancement of artificial intelligence, deep learning-based object detection methods have achieved remarkable success in visual inspection tasks. Existing methods can be broadly categorized into two types: two-stage and single-stage detectors. Two-stage detectors, such as Faster R-CNN and Mask R-CNN, typically achieve high detection accuracy by decoupling localisation and classification tasks. For example, Zhang et al. [4] proposed a Sequence Transduction method that detects insulator defects by comparing differences between defective and normal regions. Fu et al. [5] introduced I2D-Net, which integrates multi-path feature fusion with a context-aware module to enhance defect representation. However, the sequential processing pipeline of two-stage methods often leads to relatively low inference efficiency.

In contrast, single-stage detectors, represented by the YOLO series, perform localisation and classification simultaneously, enabling faster inference. Hu et al. [6] proposed SGFP-YOLO, which incorporates a state space model to enhance feature representation under complex backgrounds. Yu et al. [7] developed an improved YOLO-based method for infrared insulator detection, introducing directional bounding boxes and probabilistic IoU loss to improve detection of rotated objects. Despite these advancements, detecting insulator defects remains challenging due to their small scale, diverse appearance, and interference from complex backgrounds.

To address these challenges, this paper proposes a novel detection framework termed RDA-YOLO, built upon the YOLOv8 architecture. By considering the physical characteristics of insulators and the multi-scale nature of defect patterns, the proposed method aims to achieve accurate and robust detection under complex conditions. The main contributions of this work are summarised as follows:

(1) The iLSK module was designed to combine efficient inverse residuals with dynamic large kernels, decomposing spatial features into multi-scale kernels. This enables the network to adaptively adjust its receptive field for detecting objects of varying sizes.
(2) The DyHead architecture was introduced to replace conventional detection heads, integrating attention mechanisms across different dimensions. This effectively mitigates conflicts between localisation and classification tasks, enabling robust detection of objects with significant size variations.
(3) The DA-WIoU loss function was devised, modelling bounding boxes as two-dimensional Gaussian distributions. By incorporating normalised Wasserstein distance into the dynamic focusing mechanism, rigid geometric overlaps were transformed into probabilistic correspondences, significantly enhancing localisation accuracy for minute defects.

## Related work

### Deep learning-based insulator defect detection

In the field of insulator defect detection, deep learning-based object detection algorithms have gradually gained prominence due to their superior feature representation capabilities compared to traditional detection methods. These techniques are typically categorised into two types: two-stage detection and single-stage detection.

Initial research primarily focused on two-stage detection algorithms, such as Faster R-CNN and Mask R-CNN, which prioritise detection accuracy. Tan et al. [8] combined Mask R-CNN with multi-feature fusion and K-means clustering to construct a system that effectively enhances the detection accuracy of defects such as insulator flashover. Zheng et al. [9] designed a cascaded architecture incorporating sequential object localisation, super-resolution reconstruction, and defect segmentation to improve feature resolution for small targets. Wang et al. [10] proposed a cross-domain multi-level feature adaptive alignment method based on R-CNN, successfully achieving transfer from synthetic to real data domains through instance-level and global-level feature alignment. Although these methods achieve high accuracy in all situations, the complicated multi-stage pipeline leads to high computational complexity, which is difficult to meet the real-time requirements of industrial inspections.

On the other hand, YOLO-like single-stage detectors have gradually become one of the research hotspots because of their good detection speed and accuracy trade-off. Yang et al. [11] adopted Focal Loss and SIoU losses to further improve YOLOv7’s localization ability on small targets under complex scenes. Zhang et al. [12] designed a channel attention mechanism based on an improved version of YOLOv5 to reduce the low-visibility caused by fog accumulation. Hao et al. [13] proposed a novel network architecture named ID-YOLO, which introduces CSP-ResNet and bidirectional feature pyramid to mine fine-grained features efficiently for extracting small defects features. Lu et al. [14] designed CMYOLOv7 by fusing the hybrid spatial pyramid and CARAFE upsampling operator to improve the perception of small textures while maintaining detailed information during the downsampling process. Considering the diversity of insulator strings in geometric shapes. You et al. [15] developed YOLOv7 to achieve variable convolutions and variable-angle bounding boxes, enabling the network to handle insulator strings at arbitrary angles and arrangements.

With the powerful global feature modelling ability of Transformers, the Transformer series models applied to object detection have been widely studied recently. Xu et al. [16] designed Multi-Scale Feature Fusion Transformer (MSFFT), which introduces a hybrid multi-head attention module to alleviate the problem of small defect features cannot be extracted well enough. Besides. Cheng et al. [17] put forward a method called AdIn-DETR for end-to-end real-time detection. Gaussian saliency prior and predictive adapter are designed to make it converge better on small-scale weight classes. It reaches ultra-high inference speed while surpassing the original state-of-the-art YOLO.

As described above, scholars have applied deep learning and YOLO series methods to detect insulators and defects effectively. Inspired by more realistic background clutter and small target features problems, our proposed RDA-YOLO algorithm focuses on the problems of real insulators with large scales of variation. We propose an improved algorithm named iLSK attention mechanism to solve this problem and verify it by experiments.

### Applications of attention mechanisms

Attention mechanisms have been applied widely in deep learning based object detection in recent years. They learn to assign different weights to effective features dynamically. The mainstream focus is currently on channel attention and spatial attention recalibration. SE-Net [18], CBAM [19] have become representative works that model inter-dependencies between channels and positions to recalibrate feature responses. Coordinate Attention (CA) [20] encodes positional information into the network to obtain long-range dependency information. However, they use very few receptive fields, which leads to sub-optimal detection performance for targets with extreme aspect ratios.

To overcome this limitation, recent research has explored employing CNNs to emulate the large receptive fields of Transformers. RepLKNet [21] demonstrated that enlarging convolution kernel sizes significantly enhances performance, albeit at increased computational cost. To balance efficiency and effective receptive field (ERF), Visual Attention Network (VAN) [22] proposed a decomposition strategy using large kernel attention (LKA) to capture long-range contextual information. LSKNet [23] introduced a spatial selection mechanism capable of dynamically adjusting the receptive field, proving highly effective for remote sensing targets.

Beyond spatial and channel dimensions, handling the drastic scale variations in UAV imagery requires a unified perspective. SimAM [24] proposed an energy-function-based, parameter-free attention mechanism to optimise features within a 3D weight space. However, for complex multi-task learning (classification and localisation), independent attention mechanisms often fail to synergise. DyHead [25] proposes a unified attention framework integrating scale-aware, spatial-aware, and task-aware attention. By dynamically creating a unified attention space, DyHead effectively bridges the semantic gap between different feature levels.

### Loss function

Bounding box regression forms the cornerstone of object detection performance. Early approaches primarily relied on IoU (Intersection over Union) loss. However, standard IoU suffers from vanishing gradients when predicted boxes fail to overlap with ground truth boxes. To mitigate this issue, GIoU [26] introduced a penalty term based on the minimum bounding rectangle. Subsequently, DIoU [27] and CIoU further incorporated centroid distance and aspect ratio consistency to accelerate convergence. More recently, SIoU [28] introduced an angle-aware penalty to address object orientation. On regular objects, these geometry-aware losses work great. However, when you target tiny objects, these metrics are extremely position-sensitive. A few pixels deviation would lead IoU degenerating to 0 with no gradients back-propagated. To reduce the sensitivity against small objects. WIoU (Wise-IoU) [29] introduced a dynamic non-monotonic focusing mechanism to suppress gradient from low-quality samples

Most existing methods still utilize the proposed WIoU as a stand-alone objective. And directly applying WIoU is still based on Euclidean distance, which is not optimal when detecting tiny and blurred defects. Therefore, in this paper, we propose DA-WIoU, which mathematically integrates a distribution-aware distance metric based on Wasser-stein distance into the focusing mechanism of WIoU for accurate regression of tiny insulator defects.

## Method

**Fig 2:**
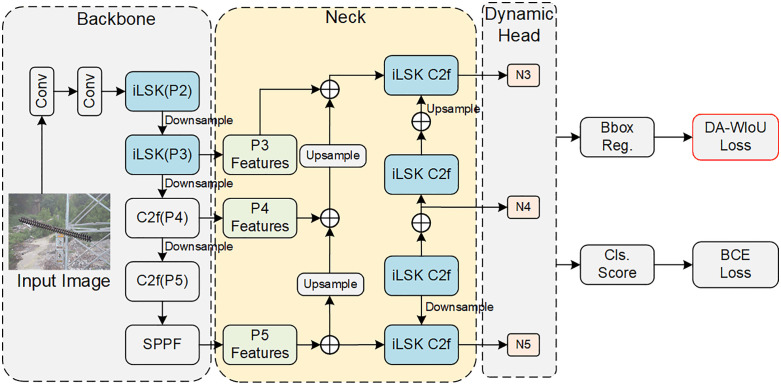
RDA-YOLO network architecture diagram. (This figure is generated based on the publicly available UPID dataset.).

The proposed RDA-YOLO framework is built upon the YOLOv8 baseline and is designed to address the challenges of multi-scale insulator defect detection in complex environments. The overall architecture is illustrated in Fig 2.

(1) To improve the representation of both global structures and fine-grained defect features, an inverted large selective kernel (iLSK) module is introduced into both the backbone and neck networks. In the backbone, the iLSK module enables adaptive receptive field selection, allowing the network to capture both large-scale contextual information (e.g., insulator strings) and small-scale defect features at an early stage. In the neck, the standard C2f module is replaced by iLSK, which enhances structural feature representation of insulators while suppressing background interference.
(2) To address the inconsistency between classification and localisation tasks in complex defect scenarios, a dynamic head (DyHead) is adopted to replace the original YOLOv8 decoupled head. The DyHead integrates scale-aware, spatial-aware, and task-aware attention mechanisms, enabling adaptive feature aggregation across different dimensions and improving detection robustness for objects with diverse scales and irregular shapes.
(3) To improve localisation accuracy for small defects, the conventional CIoU loss is replaced with the proposed distribution-aware Wasserstein IoU (DA-WIoU) loss. By modelling bounding boxes as Gaussian distributions and incorporating a normalized Wasserstein distance, the proposed loss provides a smoother optimisation objective and enhances localisation performance, particularly for small-scale targets.

### iLSK (inverted large selective kernel module)

**Fig 3:**
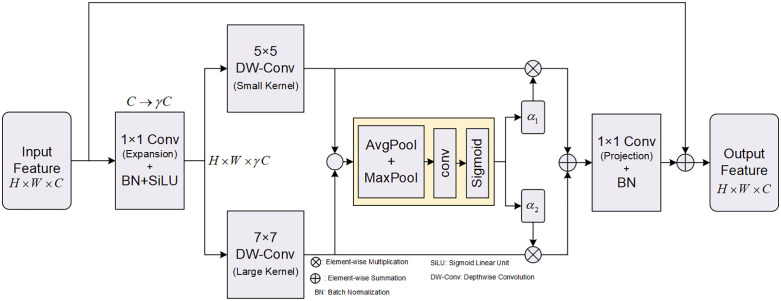
Inverted large selective kernel module architecture diagram.

The fixed convolution kernel in traditional CNN leads to a fixed receptive field for all convolution layers. However, in insulator and defect detection, there will be many insulator strings in a picture while insulators just account for a small amount of the whole picture. Large receptive fields are needed to obtain global contextual information while small receptive fields are required to capture local fine-grained features of the defects like missing insulator, breakage, flashover et al. To solve the problem, we design an iLSK module as shown in Fig 3. The module inserts the large kernel selection mechanism into the inverted residual structure so that network can dynamically change the size of the receptive field based on the scale of target. This allows it to simultaneously complete the detection of large-scale and small-scale targets. Where is the specific network structure.

The receptive field of traditional CNNs is fixed due to the static convolution kernel. In insulator and defect detection tasks, there are often many insullator strings in the picture, which account for a large percentage. To extract the global contextual information of such large targets, i.e., insulator strings, networks need large receptive fields. However, for small targets such as missing insulators, breakages, flashover and other defects, only a few pixels need to be recognized. Networks need to learn ultra-fine local features through small receptive fields to identify these small targets. At present, to capture multi-scale features of targets, most methods rely on the enlargement of the network architecture, which greatly increases the network complexity and reduces the computational efficiency. Inspired by this, we propose an iLSK module, as shown in Fig 3. iLSK integrates a large kernel selection strategy into the inverted residual structure, which can adjust the size of the receptive field of network adaptively according to the scale of the target. Realize the detection of large and small targets in compatibility. Below is a detailed introduction to our network architecture.

Assume the input feature map is $X\in R^{H\times W\times C}$. iLSK first applies pointwise convolution on X to expand the feature channel dimension, which enhances the representational ability of non-linear fitting. The process is defined as follows:


$$\begin{array}{c} \tilde{X}=\phi \left(B\left(\overset{exp}{\underset{\text{1}\times \text{1}}{F}}(X)\right)\right) \end{array}$$ (1)

Where, $\overset{exp}{\underset{\text{1}\times \text{1}}{F}}(\cdot )$ represents 1 × 1 convolution with expansion factor γ (set to 4 in this paper), which means expand the number of channels to γC; B(⋅) represents Batch Normalisation; ϕ(⋅) represents SiLU activation function.

Inspired by the zoom function of the human visual system, we then split the spatial features extracted above into two parallel groups of depth-wise convolution kernels with different kernel sizes and dilation rates:


$$\begin{array}{c} U_{\text{1}}=D_{\text{5}\times \text{5},d=\text{3}}(\tilde{X}),U_{\text{2}}=D_{\text{7}\times \text{7},d=\text{3}}(\tilde{X}) \end{array}$$ (2)

Where, $D_{k\times k,d}(\cdot )$ represents depthwise convolution with kernel size k and dilation rate d.

To further adaptively fuse the above two features, we calculate the spatial selection descriptor $S_{desc}$. Specifically, we first fuse the information of the two branches by element-wise summation, and then obtain the spatial statistical information by max pooling with average pooling kernel:


$$\begin{array}{c} \tilde{U}=U_{\text{1}}+U_{\text{2}} \end{array}$$ (3)

$$\begin{array}{c} S_{desc}=F_{conv}(\left[P_{avg}(\tilde{U});P_{max}(\tilde{U})\right]) \end{array}$$ (4)

Then we use the Sigmoid function to obtain spatial attention maps $\alpha_{\text{1}},\alpha_{\text{2}}\in R^{H\times W\times \text{1}}$:


$$\begin{array}{c} \alpha_{\text{1}}=\sigma (S_{desc}),\alpha_{\text{2}}=\text{1}-\alpha_{\text{1}} \end{array}$$ (5)

The final output of iLSK is obtained through multi-scale weighting:


$$\begin{array}{c} Y_{attn}=\alpha_{\text{1}}⊙U_{\text{1}}+\alpha_{\text{2}}⊙U_{\text{2}} \end{array}$$ (6)

Then we use linear projection to restore the dimensionality of features and add residual connections to help gradients propagate through the network:


$$\begin{array}{c} Y=\text{B}(\overset{proj}{\underset{\text{1}\times \text{1}}{F}}(Y_{attn}))+X \end{array}$$ (7)

As illustrated in Fig 3, from the perspective of network topology, the iLSK module was designed to integrate multi-scale feature extraction with dynamic receptive field selection. The core advantage of this architecture lies in the synergy of three key components:

1. The two 1 × 1 convolutions at the front and rear ends form an inverted residual structure. This approach employs a strategy of first increasing dimensionality followed by dimensionality reduction. It ensures that the intermediate feature extraction process occurs within a higher-dimensional space with greater expressive power, while simultaneously preventing an excessive number of parameters.
2. The parallel 5 × 5 and 7 × 7 convolutions function physically as micro-detail processing and macro-context perception. The former excels at extracting minute defect features such as insulator damage and flashover, while the latter facilitates better localisation of the entire insulator string.
3. The intermediate pooling and activation layers function as dynamic feature decision-making units. Rather than statically augmenting traditional network features, they dynamically output spatial weights tailored to the current region’s characteristics. When processing minute defects, the network automatically elevates weights for small kernels; conversely, when handling large insulator strings, it boosts weights for large kernels. This approach perfectly accommodates object detection tasks across extreme scale variations.

### Dynamic head

**Fig 4:**
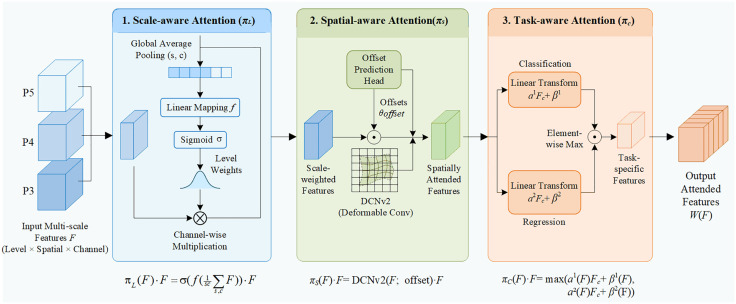
Dynamic head module architecture diagram.

As for drone aerial photographs taken of insulators and defect detection, there are generally two problems with detection head: (1)the background is complicated, which makes feature pyramid needs to deal with foreground insulator strings and tiny defects in background at the same time;(2)The feature for classification and localisation are quite different. Traditional static detection heads, based on fixed receptive fields and weights, limit adaptability. Therefore, we introduce the Dynamic Head in this paper, which unifies scale-aware, spatial-aware, and task-aware attention into a single module, enabling dynamic adjustment of their relative attentions. The specific architecture is illustrated in Fig 4.

(1) Scale-aware

The scale sensitivity component dynamically adjusts the weights of different feature levels to resolve conflicts between large and small targets.


$$\begin{array}{c} \pi_{L}(F)\cdot F=\sigma \left(f\left(\frac{\text{1}}{SC}\sum_{S,C}\;F\right)\right)\cdot F \end{array}$$ (8)

Where, f(•) is a linear mapping function, and σ(•) is the sigmoid activation function.

(2) Spatial-aware

Spatial perception can learn sparse spatial position weights, aggregating spatial information across levels.


$$\begin{array}{c} \pi_{spatial}(\text{F})\cdot \text{F}=\sum_{k=\text{1}}^{K}\;w_{k}\cdot \text{F}(p+\Delta p_{k})\cdot \Delta m_{k} \end{array}$$ (9)

Where DCNv2(•) denotes deformable convolution.

(3) Task-aware

Task-aware approaches dynamically adjust channel responses, as object detection primarily involves two tasks: classification and regression. This decouples the task conflicts between them.


$$\begin{array}{c} \pi_{C}(F)\cdot F=max(\alpha^{\text{1}}(F)F_{c}+\beta^{\text{1}}(F),\alpha^{\text{2}}(F)F_{c}+\beta^{\text{2}}(F)) \end{array}$$ (10)

As illustrated in Fig 4, the Dynamic Head module performs a deep reconstruction of the input feature pyramid from features to tasks and from micro to macro levels by cascading three-dimensional attention mechanisms. This process comprises three crucial evolutionary stages:

1. Scale Awareness: By aggregating global context to dynamically adjust weights across feature layers, the network focuses on appropriate feature hierarchies according to the target’s actual size. This mitigates feature conflicts arising from extreme scale disparities.
2. Spatial Awareness: Employing deformable convolutions as the primary module for dynamic receptive field regulation, it effectively guides features towards genuine target characteristics amidst complex aerial background interference, thereby enabling collective perception of irregular defects.
3. Task-Awareness: Recognising that classification and localisation tasks demand distinct feature responses, this module employs parallel dynamic linear transformations to provide optimal feature representations for both tasks.

This tripartite aggregation architecture overcomes the limitations of traditional static detection heads, significantly enhancing aerial power line inspection performance in complex backgrounds.

### DA-WIoU loss function

**Fig 5:**
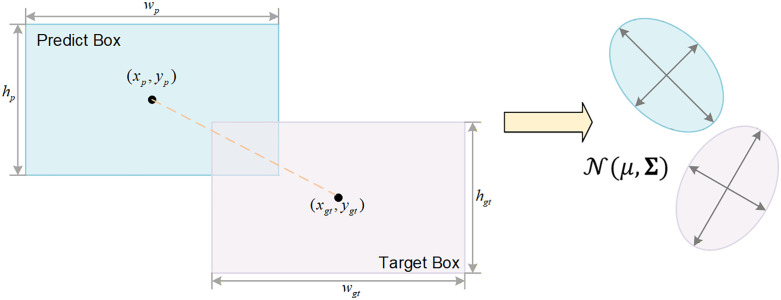
Gaussian modelling process for the DA-WIoU loss function.

The traditional IoU loss function suffers from vanishing gradient issues when handling small objects. When the predicted bounding box does not overlap with the ground truth box, the IoU is zero, leading to missed detections. To address this, WIoU introduced a dynamic focus mechanism. However, its metric based on Euclidean distance is overly sensitive to detection offsets for small objects, potentially causing training oscillations. We therefore propose the DA-WIoU loss function, which models bounding boxes as a two-dimensional Gaussian distribution. It incorporates the Wasserstein distance to represent geometric distance, defining similarity metrics for bounding boxes from a probabilistic distribution perspective. The specific transformation is illustrated in Fig 5.

For the bounding box B=(cx,cy,w,h), where (cx,cy) denotes the centre point coordinates and (w,h) represents the width and height, we model it as a two-dimensional Gaussian distribution N(μ,Σ):


$$\begin{array}{c} \mu =\left[\begin{array}{c} cx\\ cy \end{array}\right],\Sigma =[\begin{array}{c} \;\;\;\;\frac{w^{2}}{4}\;0\\ 0\;\frac{h^{2}}{4} \end{array}] \end{array}$$ (11)

The discrepancy between the predicted box distribution and the ground truth box distribution is measured using the second-order Wasserstein distance, defined by the formula:


$$\begin{array}{c} \overset{\text{2}}{\underset{\text{2}}{W}}=(cx_{p}-cx_{gt}{)}^{\text{2}}+(cy_{p}-cy_{gt}{)}^{\text{2}}+\frac{(w_{p}-w_{gt}{)}^{\text{2}}+(h_{p}-h_{gt}{)}^{\text{2}}}{\text{4}} \end{array}$$ (12)

Embed this distance into the loss function, defining the normalised distribution-aware penalty term $R_{Wass}$:


$$\begin{array}{c} R_{Wass}=\text{exp}\left(\frac{\overset{\text{2}}{\underset{\text{2}}{W}}}{C^{\text{2}}}\right) \end{array}$$ (13)

Where C is a normalisation constant specific to the dataset, set to 12.8 throughout this study. This ensures that even when two boxes do not overlap at all, $R_{Wass}$ provides a smooth and continuous gradient.

Simultaneously, we retain WIoU’s dynamic focus mechanism to address the issue of sample imbalance between easy and difficult instances, defining the outlier degree β of anchor boxes as:


$$\begin{array}{c} \beta =\frac{\overset{―}{L_{IoU}}}{L_{IoU}}\in [\text{0},+\infty ) \end{array}$$ (14)

Where, $L_{IoU}=\text{1}-\text{IoU}$, $\overset{―}{L_{IoU}}$ denotes the moving average of the IoU loss, and the focus coefficient r is calculated as follows:


$$\begin{array}{c} =\frac{\beta }{\delta \alpha^{\beta -\delta }} \end{array}$$ (15)

Where α and δ are hyperparameters controlling the gradient gain.

The final DA-WIoU loss function is composed of the dynamic focus coefficient, distribution-aware penalty term, and baseline IoU loss:


$$\begin{array}{c} L_{DA-WIoU}=r\times R_{Wass}\times L_{IoU} \end{array}$$ (16)

This loss function not only retains the ability to detect challenging samples but also effectively resolves the vanishing gradient problem in small object detection.

### Ethics and permits statement‌‌

This study does not involve any fieldwork conducted in protected or regulated areas. The datasets used in this research consist of publicly available data and images collected under non-restricted conditions. Therefore, no institutional or governmental permits were required for data acquisition or experimental procedures.

## Experiments and analysis

### Experimental Setup

#### Dataset.

This paper utilised two datasets: the first employed the publicly available UPID dataset, while the second utilised a self-constructed dataset combining the CPLID dataset released by the State Grid Corporation of China with images extracted from the internet [30].

**Fig 6:**
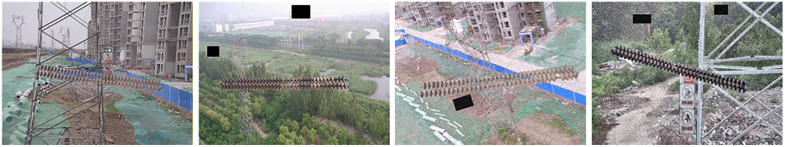
Example picture of a UPID dataset. (This figure is generated based on the publicly available UPID dataset.).

UPID dataset: Contains 6860 synthesised images of insulators and their defects, the majority of which were generated through rotation and illumination transformations applied to original images. Specific examples are shown in Fig 6.

**Fig 7:**
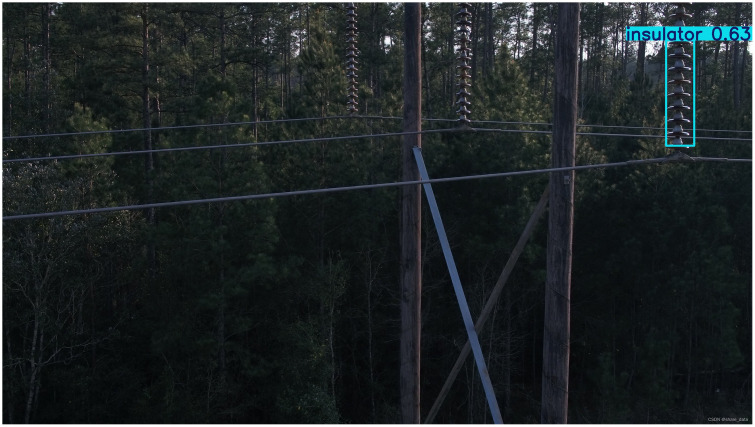
Example picture of self-built dataset.

Self-built dataset: A total of 3,000 images were collected, with 2,100 designated as the training dataset, 300 as the test dataset, and 600 as the validation dataset. All data underwent manual annotation via LabelMe, detecting four target categories: insulators, loose, flashover, and damage. Here, ‘insulators’ specifically denotes slender insulator strings, while the remaining three categories represent defects in individual insulators. Specific examples are illustrated in Fig 7.

There are some differences between UPID dataset and our self-built dataset: imaging conditions are not exactly the same, background complexity and insulator types vary, and defect distribution is inconsistent. For example, images in UPID dataset are relatively standardized compared to our self-built dataset which contains more realistic scenarios with illumination variations, viewpoint changes, and environmental factors.

Nevertheless, the visual appearances of insulator defects, such as small size, low contrast, irregular shape, etc., are consistent in these two datasets. Therefore, training and evaluation can be performed on both datasets simultaneously.

Moreover, mixed data can enhance the generalization capability of the model. As our proposed RDA-YOLO can learn generalized multi-scale and context-aware features, it can bridge the domain gaps between different datasets and still achieve robust performance in various settings.

In this study, small insulator defects are defined as targets that occupy a very limited spatial extent in the image. Specifically, these defects typically account for less than 1% of the total image area or have bounding box sizes smaller than 32 × 32 pixels under the standard input resolution of 640 × 640.

Such defects include missing insulator units, minor fractures, and discharge-related surface marks, which are often characterized by small scale, low contrast, and irregular shapes. These characteristics make them particularly challenging for detection algorithms.

#### Evaluation metrics.

Here, we employ precision, recall, mAP0.5, parameter count, and frame rate to rigorously assess model performance. Specifically, TP denotes the number of correctly detected positive samples, FN represents the number of missed positive samples, and FP indicates the number of samples incorrectly classified as positive. Precision is defined as the ratio of correctly predicted positive samples to the total number of samples classified as positive, as detailed in Equation 17. Recall is defined as the ratio of correctly predicted positive samples to the actual number of positive samples, as detailed in Equation 18. AP represents the average area under the precision-recall curve for each category, as detailed in Equation 19. mAP denotes the average of all category AP values, as detailed in Formula 20. Frame rate measures the number of frames processed per second during image or video processing, with the parameter representing the memory consumption of model parameters.


$$\begin{array}{c} \text{Precision}=\frac{\text{TP}}{\text{TP}+\text{FP}} \end{array}$$ (17)

$$\begin{array}{c} \text{Recall}=\frac{\text{TP}}{\text{TP}+\text{FN}} \end{array}$$ (18)

$$\begin{array}{c} AP=\frac{TP+TN}{TP+TN+FP} \end{array}$$ (19)

$$\begin{array}{c} mAP=\frac{\sum AP}{N} \end{array}$$ (20)

All experiments were conducted on the same experimental platform, with the specific hardware and software details outlined in Table 1. Throughout all experiments in this paper, the initial learning rate for the network was uniformly set at 0.001.

**Table 1:** Experimental environment.

Component	Model/Specifications
CPU	13th Intel Core i7-13700KF
RAM	32G
GPU	NVIDA GeForce RTX 4070 12GB
Programing Language	Python3.8
Deep learning Framework	PyTorch 2.3.1
CUDA	11.8

### Compared with current mainstream object detection methods on self-bulit dataset

To evaluate the overall performance of our proposed method, we conducted comparative analyses against current mainstream object detection algorithms on our self-built dataset. These included two-stage algorithms Faster-RCNN and SSD, single-stage algorithms YOLOv5, YOLOv8, YOLOv9, YOLOv10, and the transformer-based RT-DETR model.

The experimental results are presented in Table 2. Specifically, our approach demonstrates superior detection accuracy compared to other algorithms, particularly against the relatively advanced RT-DETR model. RT-DETR, while having higher precision, recall, and AP than previous deep leaning algorithms, uses a more complex transformer backbone. Leading to much higher parameter counts and slower inference speeds. Our method sees improvements in precision, recall, and mAP while keeping parameter counts low and improving inference speeds. On top of the base YOLOv8 algorithm we see gains of +1.9% precision, +6.9% recall, +4.4% mAP0.5. Adding DyHead leads to additional parameters and −1.3 fps.

**Table 2:** Comparison with mainstream object detection methods on self-built datasets.

Method	Precision	Recall	mAP0.5	Params/MB	FPS(frame/s)
Faster R-CNN [31]	0.883	0.780	0.837	40.8	12.5
SSD [32]	0.886	0.791	0.843	28.5	30.3
YOLOv5 [33]	0.890	0.743	0.822	39.5	13.4
YOLOv8(baseline) [34]	0.897	0.809	0.870	30.0	27.5
YOLOv9 [35]	0.873	0.807	0.867	32.5	23.3
YOLOv10 [36]	0.879	0.831	0.869	36.7	18.6
RT-DETR [37]	0.902	0.845	0.886	48.6	8.2
Ours	**0.916**	**0.878**	**0.914**	31.7	26.2

**Fig 8:**
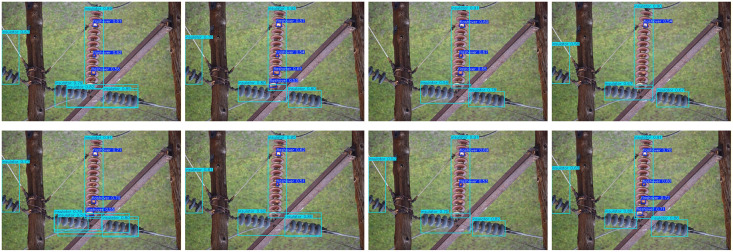
Insulator defect detection under grass background. (This figure is generated based on the publicly available UPID dataset.).

**Fig 9:**
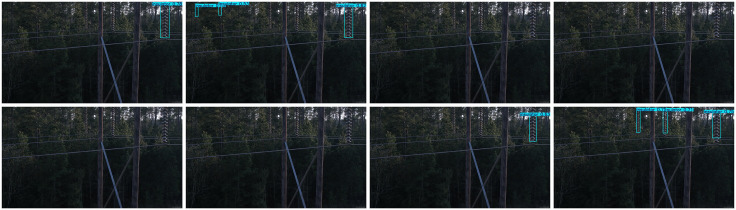
Insulator defect detection under low-light and vertical tree interference. (This figure is generated based on the publicly available UPID dataset.).

**Fig 10:**
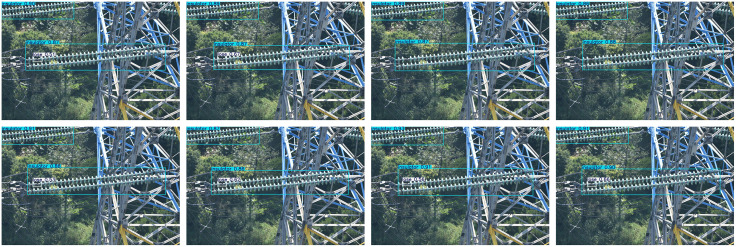
Insulator defect detection under occluded background. (This figure is generated based on the publicly available UPID dataset.).

Detection was also performed on various backgrounds, including high contrast grass background, low light backgrounds with vertical tree disturbance, and cluttered backgrounds with occlusions. Images of these detections can be found in Figs 8–10. The methods are presented in the following order: Faster R-CNN, SSD, YOLOv5, YOLOv8 (Baseline), YOLOv9, YOLOv10, RT-DET, and Ours.

Figure 8 showns examples compared to high contrast grass background. As shown in Fig 6, all algorithms were able to detect the obvious string of insulators. But differed when it came to strings being obscured. Algorithms such as Faster R-CNN, YOLOv5, YOLOv9, and YOLOv10 showed multiple detections for false positives. While our algorithm was able to remove background noise from within obscured insulator strings to detect properly. Baseline algorithms also had significant issues with false positives on small targets such as flashovers. Which are small, low contrast targets. Our improvement was able to detect all examples of defect state with significantly higher confidence scores showing Dyhead is able to better resolve conflicts between detection versus localisation.

As shown in Fig 9, simpler single stage algorithms were unable to correctly localise insulators and produce a detection. Faster RCNN and RT-DETR were able to find parts of the insulator string but missed significant amounts. SSD was unable to differentiate between background trees. Our proposed method, despite some non-overlapping bounding boxes, successfully localised and detected all insulators without false positives or false negatives.

Figure 10 illustrates more complex backgrounds and occlusion scenarios. It is evident that baseline models failed to detect missing insulators due to similar target textures and backgrounds. Our method achieved the highest detection accuracy among all approaches.

**Fig 11:**
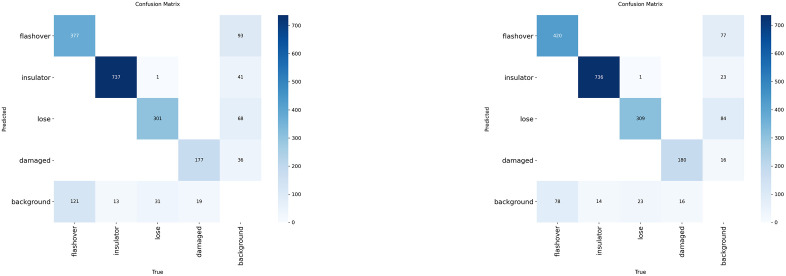
Comparison of confusion matrices between the baseline model (YOLOv8) and the improved model (RDA-YOLO) on the test set.

Figure 11 presents a comparison of the confusion matrices between the baseline model YOLOv8 and the improved model RDA-YOLO. Compared to the baseline model, RDA-YOLO demonstrates a significant improvement in recognition accuracy across all categories of insulator defects. This is particularly evident for the challenging flashover category, where the number of correctly predicted instances surged from 377 to 420. More critically, the model substantially reduced the number of missed samples where ‘flashover’ was misclassified as background from 121 to 78, whilst also markedly decreasing the background false alarm rate. This result intuitively shows that with the help of iLSK and DA-WIoU, we made the model qualitatively stronger at depicting finer details of defects and being robust to background noise.

### Compared with current mainstream object detection methods on UPID dataset

To further evaluate the generalisation capability and robustness of our proposed RDA-YOLO algorithm, particularly regarding its performance on images with varying data distributions, we conducted cross-validation on the UPID dataset. In this experiment, we compare the RDA-YOLO algorithm against state-of-the-art single-stage and two-stage approaches. To more intuitively demonstrate our algorithm’s superiority in detecting minute features, we introduce Small Object Average Precision alongside the aforementioned evaluation metrics. The results are presented in Table 3.

**Table 3:** Comparison with mainstream object detection methods on the public UPID dataset.

Model	Precision	Recall	mAP0.5	${AP}_{S}$	Params/MB	FPS(frame/s)
Faster R-CNN [2]	0.852	0.741	0.812	0.453	40.8	12.5
SSD [3]	0.861	0.755	0.818	0.421	**28.5**	**30.3**
YOLOv5 [4]	0.874	0.728	0.805	0.495	39.5	13.4
YOLOv8(baseline) [5]	0.881	0.782	0.852	0.521	30.0	27.5
YOLOv9 [6]	0.855	0.793	0.848	0.536	32.5	23.3
YOLOv10 [7]	0.868	0.805	0.856	0.548	36.7	18.6
RT-DETR [8]	0.890	0.818	0.868	0.572	48.6	8.2
Ours	**0.903**	**0.851**	**0.895**	**0.614**	31.7	26.2

As illustrated in Table 3, since UPID dataset contains more complex distribution of domains, all the models suffer from a performance drop to some extent. However, it can be seen that our proposed RDA-YOLO always achieves better performance than the competing methods in all evaluation metrics.

Compared with baseline YOLOv8, our proposed method achieves 4.3% improvement in mAP@0.5, which shows that the proposed method has obvious advantages in detecting small objects. This also further proves the superiority of our proposed modules in improving the representation ability of multi-scale features.YOLO and SSD-based methods lose fine-grained features of small defects caused by multiple downsampling operations, leading to false-negative during detection. Transformer-based RT-DETR model can improve accuracy (0.890) by mining global contextual correlation through self-attention mechanism, but its extremely high computational complexity and slow inference speed restrict its use on edge devices with limited computing resources such as UAV.

Therefore, our proposed RDA-YOLO obtains higher detection accuracy with lower computational consumption, which is more suitable for practical smart grid inspection scenarios.

It should be noted that all models are trained with the same hardware and the same input resolution to ensure fair comparisons. Therefore, the performance improvement comes from the difference in model design.

### Ablation studies

To validate the effectiveness of each introduced module for insulator and defect detection, ablation studies were conducted using YOLOv8 as the baseline model. In the experiments, we replaced the traditional C2f module with the iLSK module in both the Backbone and Neck networks, substituted the detection head with DyHead, and employed the DA-WIoU loss function. The ablation results are presented in Table 4. In the table, ‘√’ indicates that the corresponding component has been integrated into the network, while ‘-’ denotes the use of the corresponding module from the YOLOv8 baseline network.

**Table 4:** GP-YOLO ablation experiment results table.

Baseline	iLSK	DyHead	DA-WIoU	Precision	Recall	mAP0.5	Params/MB
√	–	–	–	0.897	0.809	0.870	30.0
√	√	–	–	0.913	0.845	0.889	30.2
√	–	√	–	0.914	0.842	0.895	30.6
√	–	–	√	0.898	0.845	0.890	30.9
√	√	√	√	**0.916**	**0.878**	**0.914**	31.7

The data in the Table 3 indicates that the baseline model achieves a mAP@0.5 of 87.0%. Upon introducing the iLSK module alone, the mAP improved to 88.9%, with recall significantly increasing from 80.9% to 84.5%. This demonstrates that the dynamic large-core mechanism effectively expands the receptive field, enhancing the model’s ability to capture the complete topological structure of insulators and reducing missed detections. Subsequently, adding DyHead alone further elevated mAP to 89.5%, indicating the unified attention mechanism excels at processing multi-scale aerial features. Moreover, merely replacing the DA-WIoU loss function also achieved 84.5% recall, demonstrating that the Wasserstein distance-based metric effectively mitigates gradient vanishing for minute defects, thereby uncovering more challenging samples.

### Attention heatmap visualization ablation studies

**Fig 12:**
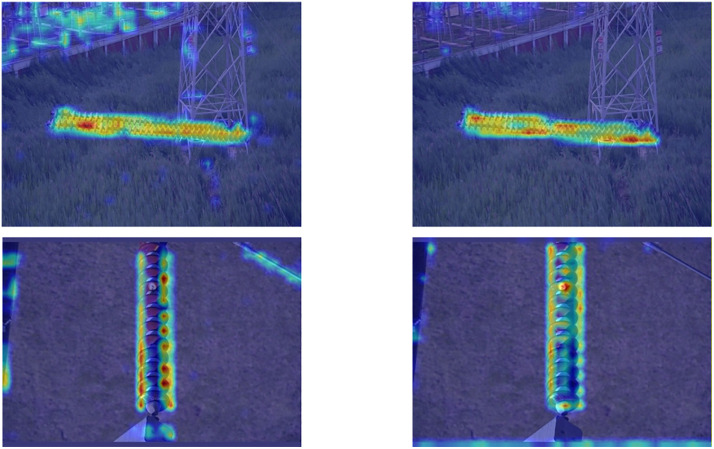
Grad-CAM heatmap visualisation comparison between baseline model (YOLOv8) and RDA-YOLO in complex aerial photography scenarios.

To more intuitively validate RDA-YOLO’s superiority in feature extraction and background suppression, we employed Grad-CAM [38] technology to visualise feature maps from the model’s output layer. Figure 12 presents heatmap comparisons between the baseline model (YOLOv8) and RDA-YOLO across varying complex scenes.

First, regarding interference resistance in the complex background of the first row, the baseline model exhibits divergent activation across the steel lattice of the tower and the grassy terrain, resulting in unfocused feature activation regions. On the contrary, the heatmap of RDA-YOLO has good focusing ability, which can suppress the back-ground noise well. This is because the unified attention mechanism of DyHead can exert good local refinement ability.

As for the localisation ability of small-target flashover defects in the second row, the baseline can locate the entire insulator string but misses the local details. Because RDA-YOLO integrates the iLSK module to adaptively adjust its receptive field. When detecting large insulator string, it can also generate high-weight activation for small-target defects. This indicates that our proposed method can alleviate the problem that small-target features are easily lost in deep networks.

### Robust detection under extreme fog conditions

In actual power grid operations, transmission lines are continuously exposed to outdoor environments, potentially encountering extreme weather conditions such as dense fog and torrential rain. These adverse conditions can significantly degrade image quality, leading to the loss of defect features. To comprehensively evaluate our proposed RDA-YOLO algorithm, we conducted experiments on our self-built dataset to simulate extreme fog interference.

**Fig 13:**
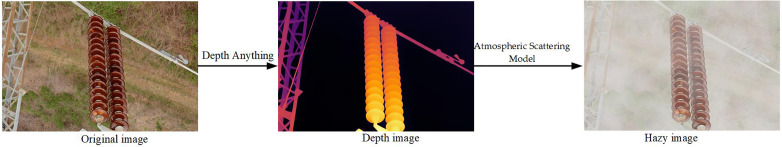
Fog Image Acquisition Process. (This figure is generated based on the publicly available UPID dataset.).


First, we employed Depth Anything technology to derive depth maps from the images [39]. Subsequently, an atmospheric scattering model (Equation 21) was applied to generate simulated fog maps. A specific example is illustrated in Fig 13.

$$\begin{array}{c} I(x)=J(x)t(x)+A(1-t(x)) \end{array}$$ (21)

Where, I(x) denotes the synthesised fog image, J(x) denotes the original image, A denotes global atmospheric illumination, and t(x) denotes the transmission map.

**Fig 14:**
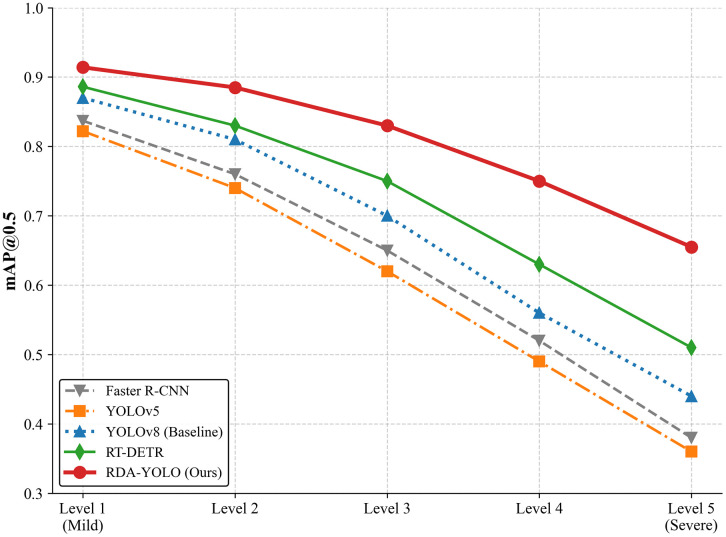
mAP drop-off curves of different detection models under varying severity levels of synthetic weather interference.

Here we categorize the dense fog into five levels, representing image degradation from slight to severe. We compare RDA-YOLO with other mainstream algorithms and obtain the mAP@0.5 descent curves under different interference intensities, as shown in Fig 14.

As can be seen from Figure 14, the detection performance of all models inevitably declines as the intensity of weather interference increases. However, the decline curves reveal that our RDA-YOLO algorithm exhibits the slowest rate of decline and demonstrates robust interference resistance. The baseline model YOLOv8 declined from an initial value of 0.870 to below 0.450. Traditional detectors also failed to maintain superior detection performance, primarily due to their static receptive fields limiting feature extraction capabilities.

In stark contrast, even at level 5 weather disturbance intensity, Our method can maintain mAP@0.5 exceed 0.650. This further demonstrates the role of our proposed iLSK module’s dynamic receptive field: when local defect features are obscured, the network leverages its expanded large kernels to acquire broader topological information, thereby achieving precise localisation. Additionally, our designed DA-WIoU loss function, by measuring bounding box similarity from a probability distribution perspective, effectively mitigates edge blurring in fog conditions.

**Fig 15:**
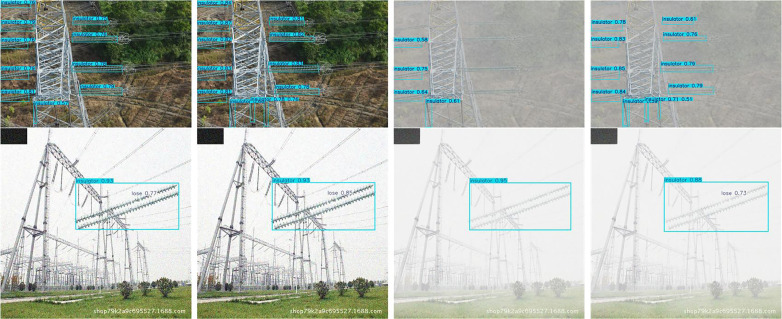
Visualisation of detection results under severe synthetic haze conditions. (This figure is generated based on the publicly available UPID dataset.).

Figure 15 presents a qualitative comparison of detection performance between the baseline model and the proposed RDA-YOLO under simulated severe fog conditions.

The results show that, under clear and high-quality imaging conditions, the proposed RDA-YOLO achieves higher confidence scores and more accurate bounding box localisation, enabling more precise and compact delineation of target regions.

However, as image quality deteriorates and visibility decreases, the performance of both models is inevitably affected. In particular, the baseline model exhibits significant degradation in feature extraction capability under dense smog conditions, leading to a substantial number of missed detections. In contrast, the proposed RDA-YOLO demonstrates stronger robustness and maintains relatively stable detection performance under such adverse conditions.

In contrast, the proposed algorithm demonstrates exceptional interference resistance and robustness. Whether confronting large-scale, complex-background insulator strings or highly challenging minute targets (insulator missing), RDA-YOLO consistently captures critical features and achieves precise detection. This comparative outcome conclusively demonstrates that our proposed algorithm effectively overcomes visual degradation issues arising from complex environments, offering superior practical value and reliability in real-world outdoor industrial inspection scenarios.

### Scale sensitivity analysis

**Fig 16:**
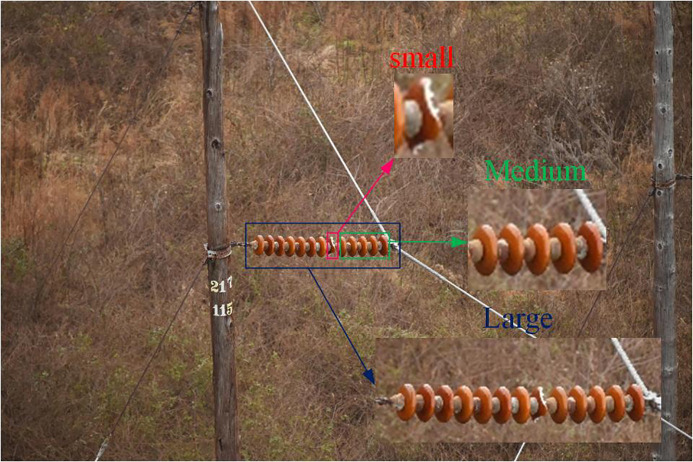
Examples of Test Sets with Different Pixel Areas. (This figure is generated based on the publicly available UPID dataset.).

To further comprehensively evaluate the robustness and generalization ability of the proposed RDA-YOLO algorithm when dealing with multi-scale targets, we strictly followed the classic MS COCO evaluation criteria and conducted scale sensitivity analysis experiments. Specifically, based on the true pixel area of the target bounding box, we finely divided the test set into three independent evaluation subsets: small targets ($area<32^{2}$), medium targets ($32^{2}<area<96^{2}$), and large targets ($area>96^{2}$). This multi-level partitioning can more objectively reflect the model’s true detection performance when handling extreme scale differences (such as tiny defects and large insulator strings). A detailed visualization example of the test set samples for the three different target scales is shown in Fig 16.

**Fig 17:**
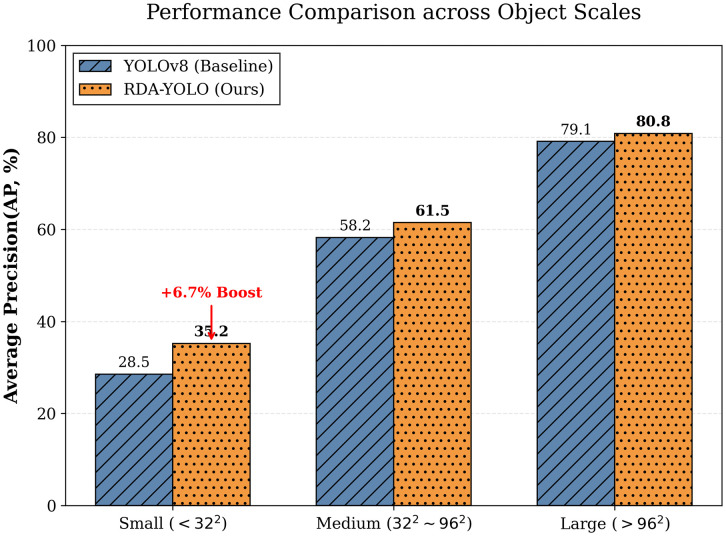
Examples of test sets with different pixel areas.

Figure 17 details the comparison of average detection accuracy between the baseline model (YOLOv8) and the proposed RDA-YOLO algorithm at three different target scales: Small, Medium, and Large.

The experimental data clearly shows that the baseline model experiences a significant drop in detection performance when dealing with small target categories. This performance bottleneck is mainly attributed to the inevitable loss of subtle high-frequency edge details and texture information of small targets during continuous subsampling in traditional networks. In contrast, RDA-YOLO demonstrates superior detection accuracy for small-scale targets, strongly validating the effectiveness of the iLSK module designed in this paper. This module successfully captures and preserves key local features of minute defects through an adaptive receptive field mechanism. Furthermore, the DA-WIoU loss function introduced in this paper plays a crucial calibration role, effectively mitigating the penalty caused by positional bias and thus significantly improving the recall rate for small target detection.

Furthermore, the comparative results further demonstrate that, in addition to its breakthrough in small target detection, RDA-YOLO has also achieved robust improvements in the detection accuracy of medium- and large-scale targets. This fully highlights the core role played by the Dynamic Head module in handling multi-scale feature deep fusion and decoupling multi-task learning, enabling the entire network architecture to maintain excellent overall detection performance under extreme scale variations.

### Sensitivity analysis of key hyperparameters

**Fig 18:**
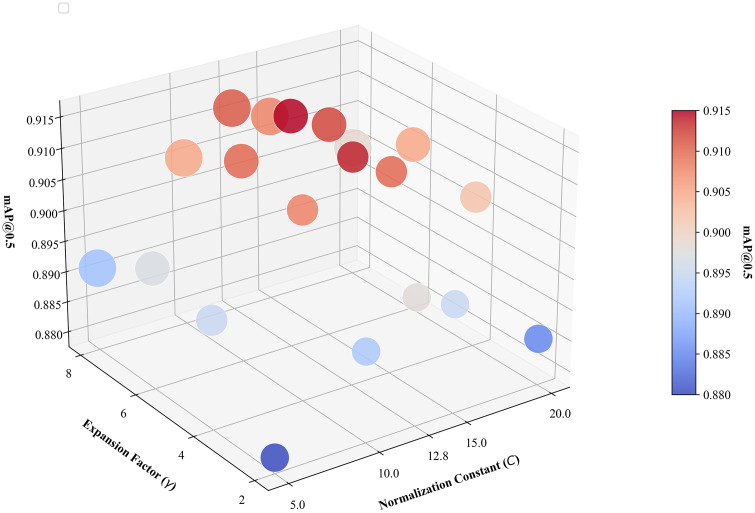
Three-dimensional scatter plot of hyperparameter sensitivity analysis. The Z-axis and colour denote mAP@0.5 in the model, while bubble size indicates the number of model parameters.

In our proposed RDA-YOLO algorithm, certain empirical hyperparameters significantly impact detection accuracy and computational complexity. To rigorously validate the rationality of our parameter selections, we conducted a grid search sensitivity analysis. We primarily focus on two hyperparameters: the normalisation parameter C in DA-WIoU (Equation 13) and the channel scaling factor γ in the iLSK module (Equation 1). For C, we selected [5, 10, 12.8, 15, 20]; for γ, we selected [2, 4, 6, 8]. Fig 18 illustrates the trade-off between detection accuracy and model parameter count.

As can be seen from Fig 18, gamma is the primary determinant of the model’s parameter count. When gamma is set to 2, the parameter count decreases to 25.1MB; however, the network’s feature representation capability diminishes at this point, resulting in a suboptimal mAP value. Conversely, when γ increases to 6 and 8, the parameter size expands to 28.4 and 30.1 MB respectively. Whilst γ=6 yields a marginal accuracy improvement over γ=4, γ=8 induces overfitting that degrades performance. After comprehensive evaluation, gamma 4 is selected for this study. Regarding the C, all gamma values corresponding to each C exhibit an inverted U-shaped distribution. Here, the empirical C of 12.8 yields the peak mAP. After comprehensive consideration, this paper selects a C of 12.8 and a gamma value of 4.

### Visualization analysis of feature space distribution

**Fig 19:**
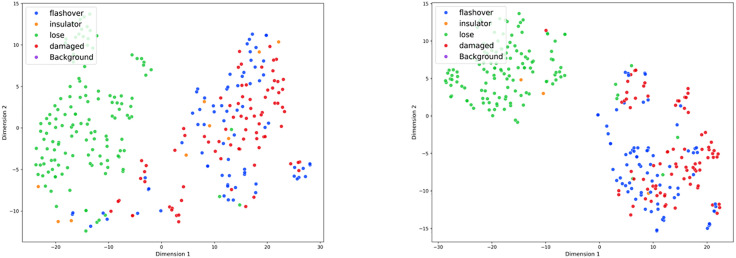
t-SNE dimensionality reduction visualisation analysis for the test set.

To analyze the benefits of RDA-YOLO in deep feature representation ability more intuitively, we further used the t-SNE algorithm to reduce the dimensionality and visualize the feature vectors obtained by the detector head. As shown in Fig 19, we visualize feature space distributions of baseline model and RDA-YOLO on test set, and scatter points with different colors represent different categories.

As shown in the visualization of Fig 19a, the features extracted by the baseline model exhibit significant dispersion and confusion in the two-dimensional space, revealing a lack of sufficient discriminative ability in its feature representation. Specifically, in the right-hand region of the feature distribution space, the feature clusters representing “flashover” and “damaged” defects show severe boundary overlap and mixing. Since these two fine-grained defects have highly similar visual appearances in real-world scenes, this high degree of overlap at the feature level makes the network prone to confusion in the final classification decision stage, leading to severe misclassification.

In contrast, Fig 19b clearly shows that the feature space mapped by the proposed RDA-YOLO algorithm exhibits superior intra-class compactness and inter-class separation. In the left-hand region of the feature space, the features of “lost” defects are highly aggregated, forming clearly defined and highly independent feature clusters, demonstrating excellent separability. More importantly, compared to the baseline model, the previously severely confused “flashover” and “damage” defect categories were effectively separated in the RDA-YOLO feature space, the overlapping regions were significantly compressed, and the distribution boundaries of the two became clearer and more distinguishable. This significant feature-level improvement is profoundly attributed to the advantages of the DyHead module in multi-task decoupling and feature perception, as well as the synergistic and complementary effect of the DA-WIoU loss function in fine-grained localization optimization. This feature visualization result, starting from the underlying logic of model representation learning, intuitively and powerfully demonstrates the superior performance and robustness of the RDA-YOLO algorithm in handling the task of detecting minute defects in complex backgrounds.

## Discussion

The RDA-YOLO model proposed in this paper demonstrates good performance in the task of insulator defect detection under complex environmental conditions; however, its applicability and limitations still require further analysis in practical engineering applications.

(1) As this method is based on image pattern recognition, its performance depends to a certain extent on the resolution and quality of the input images. Under low-resolution conditions, insulator defects often occupy only a small number of pixels, which can easily lead to the loss of feature information, thereby increasing the risk of missed detection; whereas under degraded conditions such as haze, varying lighting or motion blur, high-frequency information in the image is suppressed, causing defect boundaries to become blurred and increasing the difficulty of detection. Furthermore, irrelevant objects in complex backgrounds may bear a certain resemblance to defects in appearance, thereby introducing false positives.

To address these issues, this paper introduces an adaptive receptive field selection mechanism and a dynamic attention mechanism, thereby enhancing the model’s adaptability to multi-scale variations and complex backgrounds to a certain extent. In particular, multi-scale feature extraction helps mitigate the impact of resolution variations, whilst spatial and task-based attention mechanisms enhance the model’s focus on target regions, thereby suppressing background interference. However, under extremely low-resolution or severely degraded conditions, the model’s performance may still be subject to certain limitations.

(2) From the perspective of method generality, the RDA-YOLO model proposed in this paper does not rely on specific insulator types or single failure mechanisms, but rather models based on visual features. Consequently, this method possesses a certain degree of generalisation capability and can be extended to other types of insulation systems as well as similar small-target defect detection tasks. However, in different application scenarios, appropriate model adjustments and retraining are still required based on the specific data distribution.
(3) The method described in this paper still has certain limitations. Firstly, the model is dependent on the scale of the data and the quality of the annotations; when training data is insufficient or annotations are inaccurate, this may affect detection performance. Secondly, the proposed dynamic module increases the model’s complexity to some extent, which may impact real-time performance. Thirdly, The use of heavy fog conditions serves as a representative scenario of visual degradation commonly encountered in aerial inspection. Although it does not cover all possible environmental variations, it provides a practical and meaningful evaluation of model robustness under adverse imaging conditions. Future work will further explore additional degradation factors such as motion blur, illumination variation, and complex weather conditions. Furthermore, the current method primarily models at the visual level and has not yet incorporated additional physical information, leaving room for further expansion in future research.

In summary, the method presented in this paper demonstrates good detection capabilities in complex environments and holds potential for engineering applications; however, further optimisation is required in terms of adaptability to extreme conditions, multi-source information fusion, and lightweight design.

## Conclusion

To address the challenges of complex backgrounds, extremely small defect scales, and insufficient feature extraction capabilities of traditional detectors in UAV aerial images of insulators, this paper proposes a high-performance insulator defect detection network—RDA-YOLO. The main contributions and conclusions are summarized as follows:

1. An iLSK module was designed to achieve dynamic receptive field adjustment: This module cleverly combines an inverse residual structure with a dynamic large convolutional kernel selection mechanism. It effectively resolves the contradiction between “fitting the overall slender topology of the insulator” and “extracting local texture features of tiny defects,” achieving adaptive perception during feature extraction.
2. A DyHead dynamic detection head was introduced to optimize feature fusion: This structure integrates scale perception, spatial perception, and task-aware attention mechanisms. It not only significantly improves the model’s feature fusion and multi-scale adaptability under drastic scale changes but also fundamentally alleviates the feature conflict between classification and localization/regression tasks.
3. A DA-WIoU loss function is proposed to enhance small target regression: by incorporating the distribution-aware metric (NWD) into the WIoU focusing mechanism, this loss function effectively overcomes the gradient vanishing problem that is easily caused by extremely small targets. Simultaneously, it makes the bounding box regression process more focused, significantly reducing sensitivity to overlapping small defects.

It demonstrates excellent comprehensive detection performance and robustness in extreme environments: on a self-built insulator defect dataset, RDA-YOLO achieves 91.4% mAP@0.5, a significant improvement of 4.4% compared to the baseline model, and achieves a detection speed of 26.2 FPS, perfectly balancing the high accuracy and real-time requirements of actual smart grid inspections. Furthermore, t-SNE visualization demonstrates that the features extracted by the model possess excellent inter-class separability. Notably, robustness testing under extreme dense fog conditions further validates the model’s strong anti-interference capability in harsh weather environments.

Future research will primarily focus on the following two directions:

1. Model Lightweighting and Edge Deployment: Given the limited computing resources of UAV platforms, future research will focus on exploring model compression and lightweight design. While maintaining high detection accuracy, this will further reduce the number of network parameters and computational complexity to achieve more efficient edge migration.
2. Deep Fusion of Multimodal Data: To further overcome the limitations of visual detection in extreme weather conditions, future research will explore fusing infrared thermal imaging or depth spatial information with visible light images to achieve multimodal feature fusion, thereby comprehensively improving the model’s perception capabilities in complex, all-weather environments.

## References

[1] Cui B, Han C, Yang M, Ding L, Shuang F. DINS: a diverse insulator dataset for object detection and instance segmentation. IEEE Trans Ind Inf. 2024;20(10):12252–61. doi: 10.1109/tii.2024.3417290

[2] Luo B, Xiao J, Zhu G, Fang X, Wang J. Occluded Insulator detection system based on YOLOX of multi-scale feature fusion. IEEE Trans Power Delivery. 2024;39(2):1063–74. doi: 10.1109/tpwrd.2024.3350162

[3] Ma W, Wang B, Zhao Z, Wang Q, Chen B. A small-sized defect detection method for power line insulator using multiscale feature and lightweight networks in UAV-vision. IEEE Trans Power Delivery. 2025;40(5):2747–58. doi: 10.1109/tpwrd.2025.3589542

[4] Zhang Y, Wang B, Yang Q, Tang F, Wei K. A two-stage insulator defect detection network with sequence transduction. IEEE Trans Instrum Meas. 2025;74:1–13. doi: 10.1109/tim.2024.3522390

[5] Fu Q, Liu J, Zhang X, Zhang Y, Ou Y, Jiao R, et al. A small-sized defect detection method for overhead transmission lines based on convolutional neural networks. IEEE Trans Instrum Meas. 2023;72:1–12. doi: 10.1109/tim.2023.329842437323850

[6] Hu Z, Zhai B, Zhao Z, Zhai Y, Wang Q, Yang K. State-space-model-guided deep feature perception network for insulator defect detection in high-resolution aerial images. IEEE Trans Geosci Remote Sensing. 2025;63:1–14. doi: 10.1109/tgrs.2025.3584663

[7] Yu X, Zhou Z, Deng Y, Zhang K, Gu C, Liu Z, et al. An angle-enhanced deep learning framework for thermal defect diagnosis in overhead transmission line composite insulators. Eng Appl Artif Intell. 2025;162:112285. doi: 10.1016/j.engappai.2025.112285

[8] Tan P, Li X, Ding J, Cui Z, Ma J, Sun Y, et al. Mask R-CNN and multifeature clustering model for catenary insulator recognition and defect detection. J Zhejiang Univ Sci A. 2022;23(9):745–56. doi: 10.1631/jzus.a2100494

[9] Zheng ZQ, Zhao MY, Cheng X, Weng Z, Wang Y, editors. Defect detection for high voltage transmission lines based on deep learning. In: 5th Chinese Conference on Pattern Recognition and Computer Vision (PRCV); 2022 Nov 04–07; Shenzhen, People’s Republic of China: Southern University of Science and Technology; 2022.

[10] Wang YR, Qu Z, Hu ZD, Yang CW, Huang XG, Zhao ZB, et al. Cross-domain multilevel feature adaptive alignment R-CNN for insulator defect detection in transmission lines. IEEE Trans Instrum Meas. 2025;74.

[11] Yang Y, Yang S, Li C, Wang Y, Pi X, Lu Y, et al. Insulator defect detection under extreme weather based on synthetic weather algorithm and improved YOLOv7. High Volt. 2024;10(1):69–77. doi: 10.1049/hve2.12513

[12] Zhang ZD, Zhang B, Lan ZC, Liu HC, Li DY, Pei L, et al. FINet: an insulator dataset and detection benchmark based on synthetic fog and improved YOLOv5. IEEE Trans Instrum Meas. 2022;71.

[13] Hao K, Chen G, Zhao L, Li Z, Liu Y, Wang C. An insulator defect detection model in aerial images based on multiscale feature pyramid network. IEEE Trans Instrum Meas. 2022;71:1–12. doi: 10.1109/tim.2022.3200861

[14] Lu Q, Lin K, Yin L. 3D attention-focused pure convolutional target detection algorithm for insulator defect detection. Expert Syst Appl. 2024;249:123720. doi: 10.1016/j.eswa.2024.123720

[15] You X, Zhao X. A insulator defect detection network based on improved YOLOv7 for UAV aerial images. Measurement. 2025;253:117410. doi: 10.1016/j.measurement.2025.117410

[16] Xu J, Liao H, Li K, Jiang C, Li D. Multiscale feature fusion transformer with hybrid attention for insulator defect detection. IEEE Trans Instrum Meas. 2025;74:1–13. doi: 10.1109/tim.2025.3568984

[17] Cheng Y, Liu D. AdIn-DETR: adapting detection transformer for end-to-end real-time power line insulator defect detection. IEEE Trans Instrum Meas. 2024;73:1–11. doi: 10.1109/tim.2024.3420265

[18] Hu J, Shen L, Sun G. Squeeze-and-excitation networks. In: 2018 IEEE/CVF Conference on Computer Vision and Pattern Recognition; 2018:7132–41. doi: 10.1109/cvpr.2018.00745

[19] Woo S, Park J, Lee J-Y, Kweon IS. CBAM: convolutional block attention module. In: Lecture notes in computer science. Springer International Publishing; 2018. p. 3–19. doi: 10.1007/978-3-030-01234-2_1

[20] Yang W, Wu J, Zhang J, Gao K, Du R, Wu Z, et al. Deformable convolution and coordinate attention for fast cattle detection. Comput Electron Agric. 2023;211:108006. doi: 10.1016/j.compag.2023.108006

[21] Ding X, Zhang X, Han J, Ding G. Scaling up your kernels to 31×31: revisiting large kernel design in CNNs. In: 2022 IEEE/CVF Conference on Computer Vision and Pattern Recognition (CVPR); 2022:11953–65. doi: 10.1109/cvpr52688.2022.01166

[22] Guo M-H, Lu C-Z, Liu Z-N, Cheng M-M, Hu S-M. Visual attention network. Comp Visual Med. 2023;9(4):733–52. doi: 10.1007/s41095-023-0364-2

[23] Li Y, Li X, Dai Y, Hou Q, Liu L, Liu Y, et al. LSKNet: a foundation lightweight backbone for remote sensing. Int J Comput Vis. 2024;133(3):1410–31. doi: 10.1007/s11263-024-02247-9

[24] Yang L, Zhang RY, Li L, Xie X. Simam: A simple, parameter-free attention module for convolutional neural networks. In: International conference on machine learning; 2021:11863–74.

[25] Dai X, Chen Y, Xiao B, Chen D, Liu M, Yuan L, et al. Dynamic Head: unifying object detection heads with attentions. In: 2021 IEEE/CVF Conference on Computer Vision and Pattern Recognition (CVPR); 2021:7369–78. doi: 10.1109/cvpr46437.2021.00729

[26] Liu X, Hu J, Wang H, Zhang Z, Lu X, Sheng C, et al. Gaussian-IoU loss: better learning for bounding box regression on PCB component detection. Expert Syst Appl. 2022;190:116178. doi: 10.1016/j.eswa.2021.116178

[27] Zheng Z, Wang P, Liu W, Li J, Ye R, Ren D. Distance-IoU loss: faster and better learning for bounding box regression. AAAI. 2020;34(07):12993–3000. doi: 10.1609/aaai.v34i07.6999

[28] Gevorgyan Z. SIoU loss: more powerful learning for bounding box regression. arXiv preprint. 2022:220512740. doi: 10.48550/arXiv.220512740

[29] Tong Z, Chen Y, Xu Z, Yu R. Wise-IoU: bounding box regression loss with dynamic focusing mechanism. 2023. Available from: https://arxiv.org/abs/2301.10051

[30] Wang HZ. Insulator data set ‐ Chinese Power Line Insulator Dataset (CPLID); 2018.

[31] Ren S, He K, Girshick R, Sun J. Faster R-CNN: towards real-time object detection with region proposal networks. IEEE Trans Pattern Anal Mach Intell. 2017;39(6):1137–49. doi: 10.1109/TPAMI.2016.2577031 27295650

[32] Liu W, Anguelov D, Erhan D, Szegedy C, Reed S, Fu C-Y, et al. SSD: Single shot multibox detector. In: Lecture notes in computer science. Springer International Publishing; 2016:21–37. doi: 10.1007/978-3-319-46448-0_2

[33] Jocher G, Stoken A, Borovec J, Changyu L, Hogan A, Diaconu L. Ultralytics/yolov5: v3.0. Zenodo. 2020.

[34] Sohan M, Sai Ram T, Reddy CV. A review on yolov8 and its advancements. 2024.

[35] Wang C-Y, Yeh IH, Mark Liao H-Y, editors. Yolov9: Learning what you want to learn using programmable gradient information. In: International conference on data intelligence and cognitive informatics. 2024. pp. 529–45.

[36] Chen H, Chen K, Ding G, Han J, Lin Z, Liu L, et al. YOLOv10: real-time end-to-end object detection. Adv Neural Inf Process Syst. 2024;37:107984–8011. doi: 10.52202/079017-3429

[37] Wang S, Xia C, Lv F, Shi Y. RT-DETRv3: real-time end-to-end object detection with hierarchical dense positive supervision. WACV. 2025:1628–36.

[38] Selvaraju RR, Das A, Vedantam R, Cogswell M, Parikh D, Batra D. Grad-CAM: why did you say that? arXiv preprint. 2016. doi: 10.48550/arXiv.1611.07450

[39] Yang L, Kang B, Huang Z, Xu X, Feng J, Zhao H. Depth anything: Unleashing the power of large-scale unlabeled data. In: Proceedings of the IEEE/CVF conference on computer vision and pattern recognition. 2024. pp. 10371–81. doi: 10.1109/cvpr52733.2024.00987

